# Risk of pulmonary fungal infections associated with biologics: a FAERS database disproportionality analysis

**DOI:** 10.3389/fimmu.2025.1672343

**Published:** 2025-10-20

**Authors:** Jing Li, Zhi Wu Han, Shan Shan Gao, Qie Guo, Huai Qin Cang

**Affiliations:** Department of Pharmacy, Affiliated Hospital of Qingdao University, Qingdao, China

**Keywords:** biologics, pulmonary fungal infection, disproportionality, FDA, adverse event

## Abstract

**Objective:**

biologics have significantly advanced the treatment of rheumatoid arthritis (RA) and psoriatic arthritis (PsA). However, real-world data regarding the risks of pulmonary fungal infections (PFI) associated with different biologics are limited. Our study aimed to explore PFI incidence among approved biologics, drawing on sources of real-world evidence.

**Methods:**

We conducted a disproportionality analysis to evaluate the association between biologics and PFI using data from the Food and Drug Administration (FDA) Adverse Event Reporting System (FAERS) (2004–2024). We analyzed clinical features, co-occurring adverse events (AEs), time-to-onset (TTO).

**Results:**

Our analysis included 3,695 patients who developed PFI following treatment with biologics. The study comprised 28.5% females, 28.6% males, and 42.9% with unspecified gender. The median age was 63 years (interquartile range [IQR] 52–71). Several biologics were associated with elevated PFI risk. Among them, the highest reporting odds ratio (ROR) were observed for infliximab(ROR = 26.02, 95% CI 17.72–38.21), rituximab(ROR = 16.23, 95% CI 13.06–20.18), tocilizumab(ROR = 14.45, 95% CI 12.28–17.00), and baricitinib(ROR = 11.01, 95% CI 7.77–15.59). Other biologics associated with a disproportionality signal in PFI risk included golimumab(ROR = 6.73, 95% CI 2.15-21.13), upadacitinib(ROR = 4.61, 95% CI 2.61–8.14), ustekinumab(ROR = 4.58, 95% CI 1.46–14.36), adalimumab(ROR = 3.45, 95% CI 2.08–5.72), tofacitinib(ROR = 3.18, 95% CI 2.04–4.95), abatacept(ROR = 3.16, 95% CI 1.74–5.73), etanercept(ROR = 2.58, 95% CI 2.06–3.24), certolizumab pegol(ROR = 1.64, 95% CI 1.27–2.10). The signal for secukinumab was not statistically (ROR = 1.70, 95% CI 0.55–5.32). Female was associated with an elevated risk of PFI and fatal outcomes in the logistic regression analysis. Tocilizumab and baricitinib showed a disproportionality signal for fatal outcomes. Our analysis suggested a trend of more pronounced PFI risk signals in elderly patients. TTO analysis demonstrated no significant gender-based differences. However, significant intergroup differences were observed between patients aged 45–64 years and those aged 65–74 years. Notably, TTO profiles varied substantially among biologics, ranging from 30 days (tocilizumab) to 393 days (etanercept).

**Conclusion:**

Our findings suggest that concomitant use of biologics is associated with a stronger disproportionality signal for PFI. The inherent limitations and potential reporting biases of the FAERS database necessitate confirmation through large-scale, prospective clinical studies.

## 1 Introduction

The expanding immunocompromised patient population has rendered fungal infections a persistent public health concern. Opportunistic pathogens, including *Aspergillus* spp. (causing invasive aspergillosis), *Cryptococcus* spp. (causing cryptococcosis), *Pneumocystis jiroveci*i (causing pneumocystis pneumonia), and various endemic fungi, constitute the principal etiological agents of PFI. Although these organisms seldom cause disease in immunocompetent hosts, they frequently precipitate life-threatening invasive mycoses in immunocompromised individuals (1). According to guidelines from the Infectious Diseases Society of America (IDSA), high-risk patients include those who have undergone hematopoietic stem cell or solid organ transplantation, those with prolonged neutropenia, and those receiving high-dose corticosteroids or other immunosuppressive therapies. Critically ill patients—particularly those requiring prolonged mechanical ventilation, those with chronic obstructive pulmonary disease (COPD), or those treated in intensive care units (ICUs)—are also considered highly susceptible due to their compromised immune status (2).

PFIs have substantially contributed to the escalating incidence and mortality of invasive fungal diseases, especially in patients with profound immune compromise (1). The increasing prevalence of immunocompromised individuals has sustained fungal infections as a major public health challenge (3). Chronic pulmonary aspergillosis affects an estimated 1,837,272 individuals annually, with approximately 340,000 attributable deaths (18.5% case-fatality rate). Similarly, pneumocystis pneumonia affects an estimated 505,000 persons each year, resulting in 214,000 deaths (42.4% mortality rate) (4).

Biologics have become the standard of care for immune-mediated diseases (IMDs), such as RA and PsA, providing symptom relief and disease progression modification. Current biologic treatment options for RA and PsA comprise: anti-cytokine agents: tumor necrosis factor inhibitors (TNFi), interleukin-6 inhibitors (IL-6i), interleukin-17 inhibitors (IL-17i) and interleukin-12/23 inhibitors (IL-12/23i). Other mechanisms: Janus kinase inhibitors (JAKi), T-cell co-stimulation inhibitors (CTLA4-Ig), and B-cell depleting agents (5).

Numerous studies have demonstrated that TNFi, JAKi and non-TNF biologics significantly improve functional status and reduce disease activity in patients with RA (6–9). Additionally, TNFi, IL-17i, and JAKi have demonstrated efficacy in achieving an American College of Rheumatology (ACR) 20 response in patients with PsA (10, 11). Furthermore, ustekinumab has been shown to elicit superior responses in both the Psoriasis Area and Severity Index (PASI) and the Investigator’s Global Assessment (IGA) in specific studies (10).

Nevertheless, despite their significant clinical efficacy, these agents are associated with a spectrum of potential adverse effects, including infections, injection-site reactions, allergic reactions, hepatotoxicity, and gastrointestinal disorders.

The first documented cases of pulmonary histoplasmosis associated with infliximab and etanercept therapy were reported in 2002 (12). Subsequent investigations primarily consisted of descriptive analyses and small-scale observational studies (13–16). Blockade of TNF-α exerts a potent anti-inflammatory effect, resulting in reduced cytokine production, impaired monocyte recruitment, prevention of granuloma formation, and cell death—both apoptotic and nonapoptotic—in cells expressing TNFR. These effects can compromise the immune response against invasive fungal pathogens and increase susceptibility to fungal infection (17). However, current evidence regarding biologic-associated PFIs remains limited, predominantly comprising case reports involving TNFi, particularly infliximab and etanercept. In contrast, data are notably lacking on infection risks associated with other biologic classes, including IL-17i, IL-12/23i, JAKi and B-cell-depleting agents. Although isolated cases of PFI associated with these agents have been reported, no systematic comparisons have evaluated differential infection risks across biologic classes. Moreover, existing studies have failed to comprehensively evaluate critical clinical determinants of susceptibility, including the duration of drug exposure preceding infection onset (18). These knowledge gaps significantly impede the formulation of evidence-based prevention strategies for PFI in clinical practice.

This study draws on data from the FDA Adverse Event Reporting System (FAERS), a publicly accessible and comprehensive pharmacovigilance database. FAERS collects post-marketing safety reports submitted by healthcare professionals and consumers globally, and its extensive dataset specifically mitigates the limitations of pre-market clinical trials, which often suffer from insufficient sample sizes and brief observation periods. We utilized real-world adverse event data from the FAERS to identify biologics associated with elevated PFI risk through disproportionality analysis using the ROR. Additionally, we characterized concomitant adverse events across various biologic classes. Furthermore, we evaluated key clinical factors influencing PFI development, including sex, age-stratified risk profiles, survival outcomes (fatal vs. non-fatal), and TTO variations across biologic agents. Through comprehensive analysis of these multidimensional risk parameters, we aimed to develop evidence-based risk stratification frameworks and targeted prevention strategies for PFI in biologic-treated populations, seeking to inform clinical decision-making regarding biologic therapy administration.

## 2 Method

### 2.1 Data source

This study utilized original data spanning the first quarter (Q1) of 2004 to the fourth quarter (Q4) of 2024, obtained from the FAERS database. FAERS is a widely utilized, publicly accessible database that aggregates voluntary AEs reports submitted by healthcare professionals, consumers, and pharmaceutical manufacturers. As a cornerstone of pharmacovigilance, FAERS provides critical post-marketing safety surveillance data, enabling the detection of potential drug risks that may not be evident in controlled clinical trials. The FAERS database architecture comprises six structured data files. Specific biologics, including TNFi, JAKi, IL-6i, IL-17i IL-12/23i, B-cell depleting agents, and T-cell co-stimulation inhibitors, were identified using relevant keywords for data retrieval from the FAERS database. Detailed information regarding the specific biologics included in this analysis is provided in **Supplementary Table S1**.

The FAERS classifies adverse drug reactions utilizing Preferred Terms (PTs) as defined by the Medical Dictionary for Regulatory Activities (MedDRA). Standardized System Organ Classes (SOCs) are categorized based on etiology, anatomical site, or purpose. We utilized MedDRA (version 25.1) for systematic classification of AEs according to their respective SOC levels. PTs associated with biologic agents were analyzed within the SOC “Infections and infestations” (SOC code: 10021881) and the High-Level Group Term (HLGT) “Respiratory tract infections” (19). We identified thirteen PTs related to PFIs, with detailed PT listings provided in **Supplementary Table S2**.

### 2.2 Data processing procedure

To address duplicate CASEIDs (unique identifiers for FAERS reports), the record with the most recent FDA_DT (FDA receipt date) was retained. For records sharing identical CASEIDs and FDA_DTs, the entry with the highest PRIMARYID (a unique FAERS report identifier) was retained. All drug names were standardized to their generic (non-proprietary) names to ensure consistency. Both the reactions (REAC) and indications (INDI) fields were coded using MedDRA PTs. The TTO of PFI AEs was calculated as the interval between the initiation date of biologic therapy and the reported AE onset date. Reports containing illogical date entries (e.g., therapy start date subsequent to AE onset date) or missing date information were excluded from the analysis. The proportion of specific AE outcomes attributed to different biologic agents was calculated.

### 2.3 Signal mining

Disproportionality analysis, a key pharmacovigilance method, assesses potential drug-adverse event associations to guide subsequent clinical case evaluations. We employed the ROR to quantify the relative reporting likelihood of PFIs associated with biologic agents compared to other drugs in the FAERS database, thereby identifying potential safety signals. A 2×2 contingency table was constructed to calculate ROR values, comparing drug-adverse reaction combinations. Signal detection criteria required a minimum of 3 cases and a lower limit of the 95% confidence interval (CI) for the ROR exceeding 1 (20). PTs for PFI meeting these criteria were classified as biologic-associated AEs for subsequent analysis.

### 2.4 Clinical factors and TTO analysis

We conducted a descriptive analysis of the clinical characteristics in the screened reports of biological agent-related AEs, including sex, age, age group, country, therapy initiation date, outcome, event onset date, region, drug type, and weight group. Using univariate and multivariate logistic regression, we evaluated the association between gender, age, drug type, weight group, and the occurrence of PFI. Fatal outcomes were defined as death or life-threatening events. The time to AE onset was calculated as the interval between therapy initiation and event occurrence. For logistic regression analysis, the reference groups were defined as male gender, patients aged 0–44 years, and treatment with etanercept. Age groups were classified as 0–44 years, 45–64 years, 65–74 years, and ≥75 years. Etanercept had the highest prescription volume among the studied agents, while the number of reported adverse reactions associated with it was relatively low. Accordingly, etanercept was designated as the reference drug to assess the risk associated with other biologics.

### 2.5 Statistical analysis

We utilized the cumulative distribution function to estimate the probability of remaining event-free for the TTO of biological agent-related AEs. Between-group differences in the median TTO were assessed using the Kruskal-Wallis test for multiple group comparisons and the Mann-Whitney U test for pairwise comparisons. As all cases experienced the event of interest (including fatal outcomes occurring post-AE), no censoring or competing risk adjustments were applied. Univariate and multivariate logistic regression analyses were performed to calculate odds ratios (ORs) for assessing AE occurrence under different exposures. Cases with missing values (including sex, age, or TTO) were excluded from the respective analyses. Statistical significance was defined as a p-value < 0.05, with all tests being two-tailed. All statistical analyses and data visualizations were performed using R version 4.4.2.

## 3 Results

**Figure 1:**
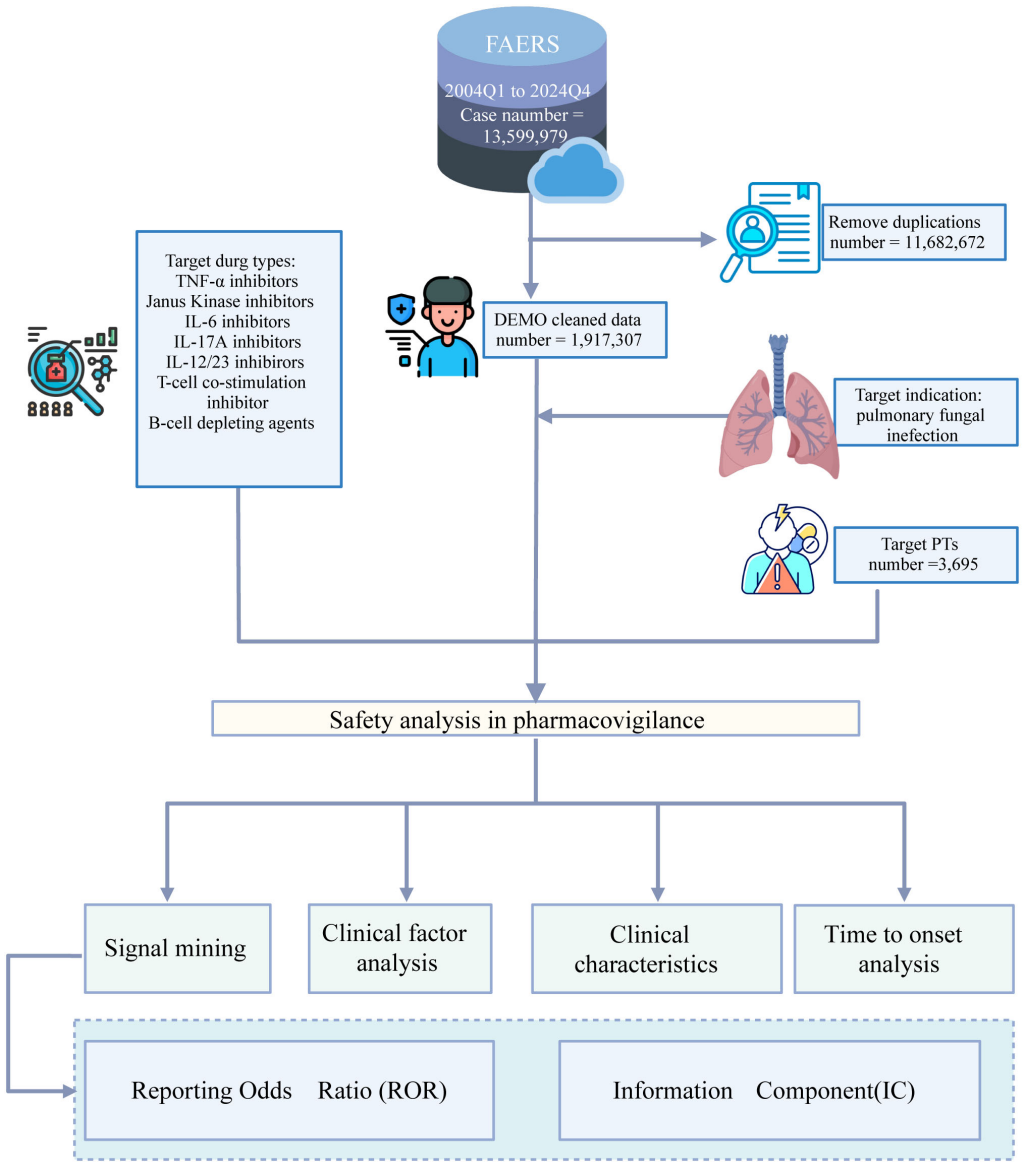
The flow chart illustrates the analytical procedure of the study.

The FAERS database contained 13,359,979 reports submitted between the first quarter of 2004 and the fourth quarter of 2024. Among these, 1,921,002 reports involved biologic agents. Following removal of duplicate records, 3965 unique reports were retained for analysis. The overall data processing workflow is depicted in **Figure 1**.

### 3.1 Demographic characteristics

We identified a total of 3,695 reports of PFI associated with the target biologics. The demographic characteristics of these cases are summarized in **Table 1**. The proportions were 28.5% female (n = 1,053), 28.6% male (n = 1,056), and 42.9% unknown gender (n = 1,265). For age groups, the proportions were: 9.4% (n = 346) for 0–44 years, 24.1% (n = 889) for 45–64 years, 20.2% (n = 748) for 65–74 years, and 12.1% (n = 447) for ≥75 years. The weight distribution was as follows: < 50 kg: 205 cases (5.5%), 50–70 kg: 401 cases (10.9%), and 70–90 kg: 241 cases (6.5%). Regarding clinical outcomes, fatal outcomes accounted for 26% (n = 961). The Region of the Americas, the Western Pacific Region, and the European Region, collectively accounting for more than 60% of the total reports. The reporting periods and case numbers were: 2004-2010: 366 cases (9.9%); 2011-2015: 729 cases (19.7%); 2016-2020: 1,120 cases (30.3%); and 2021-2024: 1,480 cases (40.1%). The distribution of cases by biological agent was: rituximab: 1,106 cases (29.9%), infliximab: 782 cases (21.2%), adalimumab: 421 cases (11.4%), tocilizumab: 388 cases (10.5%), etanercept: 292 cases (7.9%), and baricitinib: 51 cases (1.4%).

**Table 1:** The characteristics of biologics -associated pulmonary fungal infection reports submitted by FAERS database (2004 Q1–2024Q4).

Contents	Reports n(%)
Overall	3695
Sex	
Female	1053(28.5%)
Male	1056(28.6%)
Unknown	1586(42.9%)
Age group	
0-44	346 (9.4%)
45-64	889 (24.1%)
65-74	748 (20.2%)
>75	447 (12.1%)
Unknown	1265 (34.2%)
Outcome	
Fatal	961 (26.0%)
Non-Fatal	2172 (58.8%)
Unknown	562 (15.2%)
Received period	
2004-2010	366 (9.9%)
2011-2015	729 (19.7%)
2016-2020	1120 (30.3%)
2021-2024	1480 (40.1%)
Region	
Region of the Americas	921 (24.9%)
Western Pacific Region	901 (24.4%)
European Region	772 (20.9%)
African Region	9 (0.2%)
South-East Asia Region	5 (0.1%)
Unknown	1087 (29.4%)
Drug_type	
Rituximab	1106 (29.9%)
Infliximab	782 (21.2%)
Adalimumab	421 (11.4%)
Tocilizumab	388 (10.5%)
Etanercept	292 (7.9%)
Golimumab	128 (3.5%)
Abatacept	125 (3.4%)
Tofacitinib	120 (3.2%)
Certolizumab_pegol	98 (2.7%)
Upadacitinib	92 (2.5%)
Baricitinib	51 (1.4%)
Secukinumab	51 (1.4%)
Ustekinumab	41 (1.1%)
Weight_Group	
<50kg	205 (5.5%)
50-70kg	401 (10.9%)
70-90kg	241 (6.5%)
>=90kg	145 (3.9%)
Unknown	2703 (73.2%)

**Figure 2:**
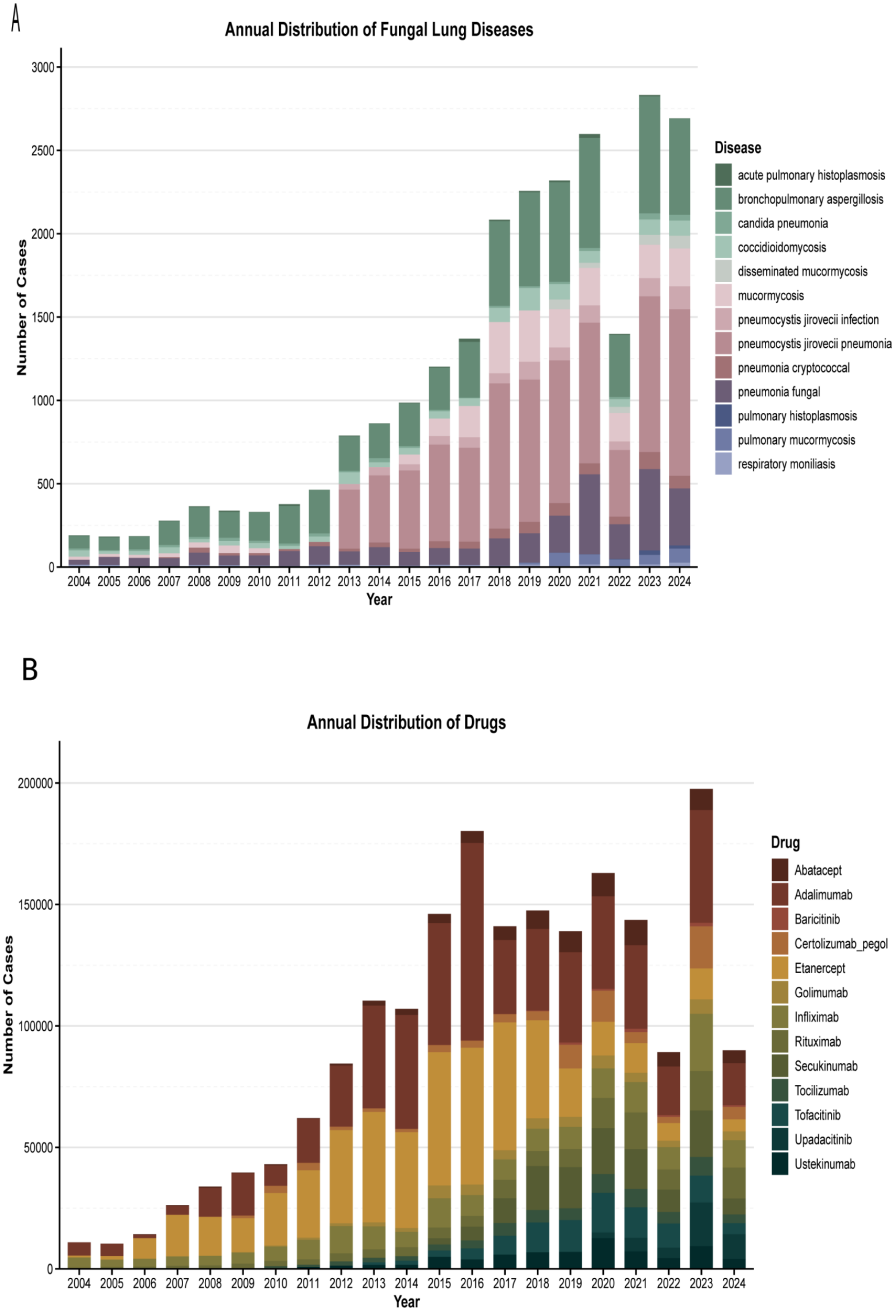
Annual Distribution of PFI and Annual Distribution of Drugs.

**Figure 2A** presents the number of patients treated with different biologics. From 2004 (dominated by infliximab and adalimumab) to 2023 (the peak reporting year), biological agent-associated PFI demonstrated a consistent upward trend (**Figure 2B**), with the exception of 2022. Pneumocystis jirovecii pneumonia (PJP; showing an increasing trend since 2013) was the most prevalent, followed by bronchopulmonary aspergillosis. The distribution of specific fungal infections was as follows: pneumocystis jirovecii infection/PJP (n = 1,513; 40.95%), aspergillosis (including fungal pneumonia and bronchopulmonary aspergillosis; n = 937; 25.36%), and coccidioidomycosis (n = 396; 10.72%).

### 3.2 Scanning for biologics -related PFI AEs

**Figure 3:**
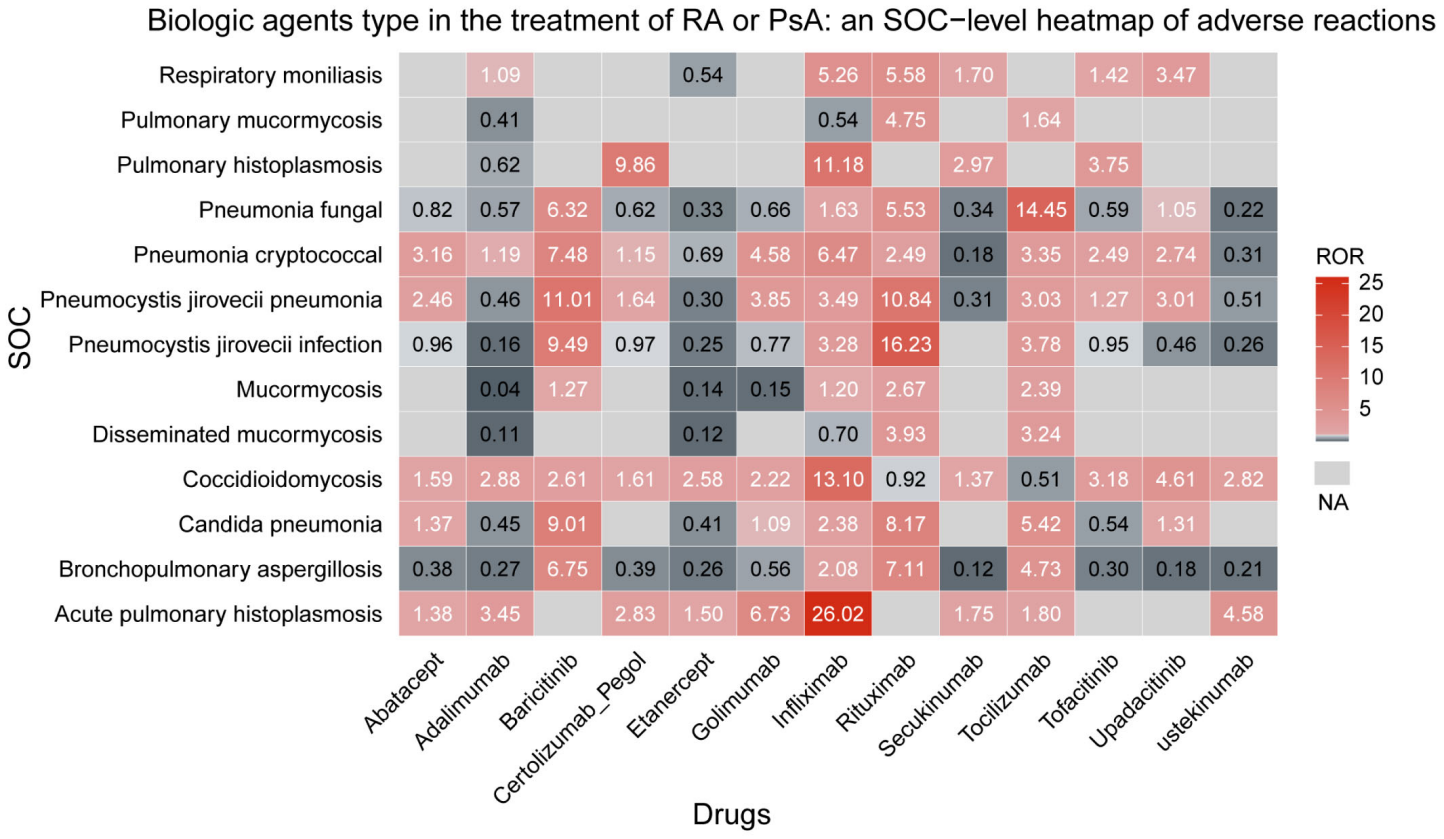
Biological agents type in the treatment of RA or PsA an SOC-level heatmap of adverse reactions.

An overall trend linking biologics to PFIs was observed, with the profile of risk differing among agents. Infliximab presented the most notable profile, being associated with the greatest number of PFI types (11) and the strongest association signal for: acute pulmonary histoplasmosis (ROR = 26.02, 95% CI 17.72–38.21), coccidioidomycosis (ROR = 13.10, 95% CI 10.93–15.70), and pulmonary histoplasmosis (ROR = 11.18, 95% CI 4.40–28.39). Rituximab was linked to10 types of PFI. for the strongest association signals were observed forpneumocystis jirovecii infection (ROR = 16.23, 95% CI 13.06–20.18) and candida pneumonia (ROR = 8.17, 95% CI 5.01–13.33). Among the 10 types of PFI associated with tocilizumab, the most pronounced association signal was for fungal pneumonia (ROR = 14.45, 95% CI 12.28–17.00). Baricitinib was associated with 8 types of PFI, with more prominent sigals identified for pneumocystis jirovecii pneumonia (ROR = 11.01, 95% CI 7.77–15.59) and bronchopulmonary aspergillosis (ROR = 6.75, 95% CI 4.13–11.04). These results are presented in **Figure 3**.

### 3.3 The accompanying AEs

**Figure 4:**
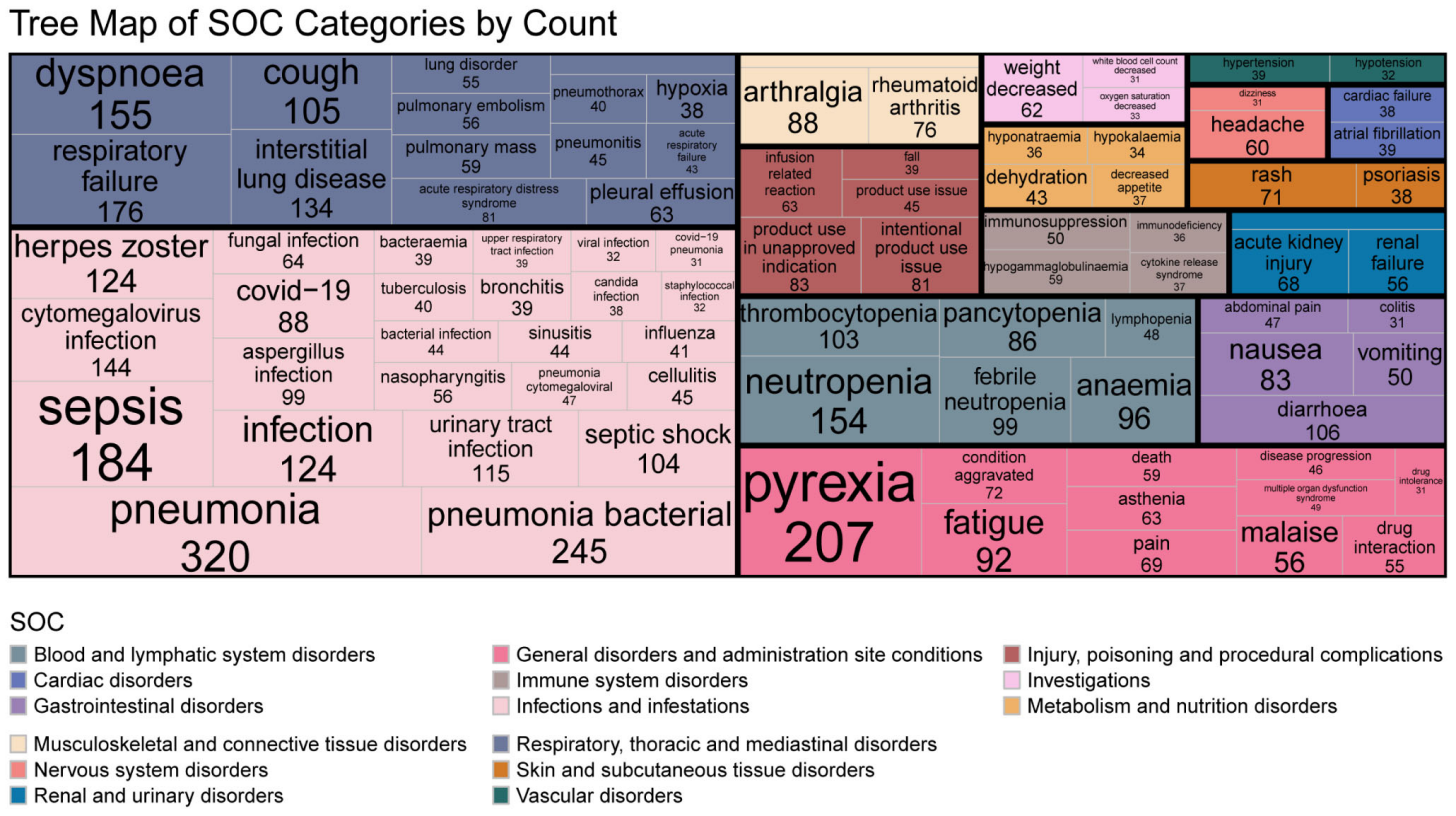
Tree Map of SOC Categories by Count(The accompanying AE of biological agents.

The most frequently reported concomitant AEs (top 30 PTs) were categorized into the following SOCs: 1) Infections and infestations: pneumonia (n = 320), bacterial pneumonia (n = 245), sepsis (n = 184), cytomegalovirus infection (n = 144), herpes zoster (n = 124), urinary tract infection (n = 115), and COVID-19 (n = 88); 2) Respiratory, thoracic and mediastinal disorders: respiratory failure (n = 176), dyspnea (n = 155), cough (n = 105), septic shock (n = 104), and acute respiratory distress syndrome (n = 81); 3) Blood and lymphatic system disorders; 4) Musculoskeletal and connective tissue disorders; 5) General disorders and administration site conditions; 6) Injury, poisoning and procedural complications; 7) Skin and subcutaneous tissue disorders; and 8) Gastrointestinal disorders. Complete details of accompaying AEs presented in **Figure 4**.

### 3.4 Clinical factors analysis

**Figure 5:**
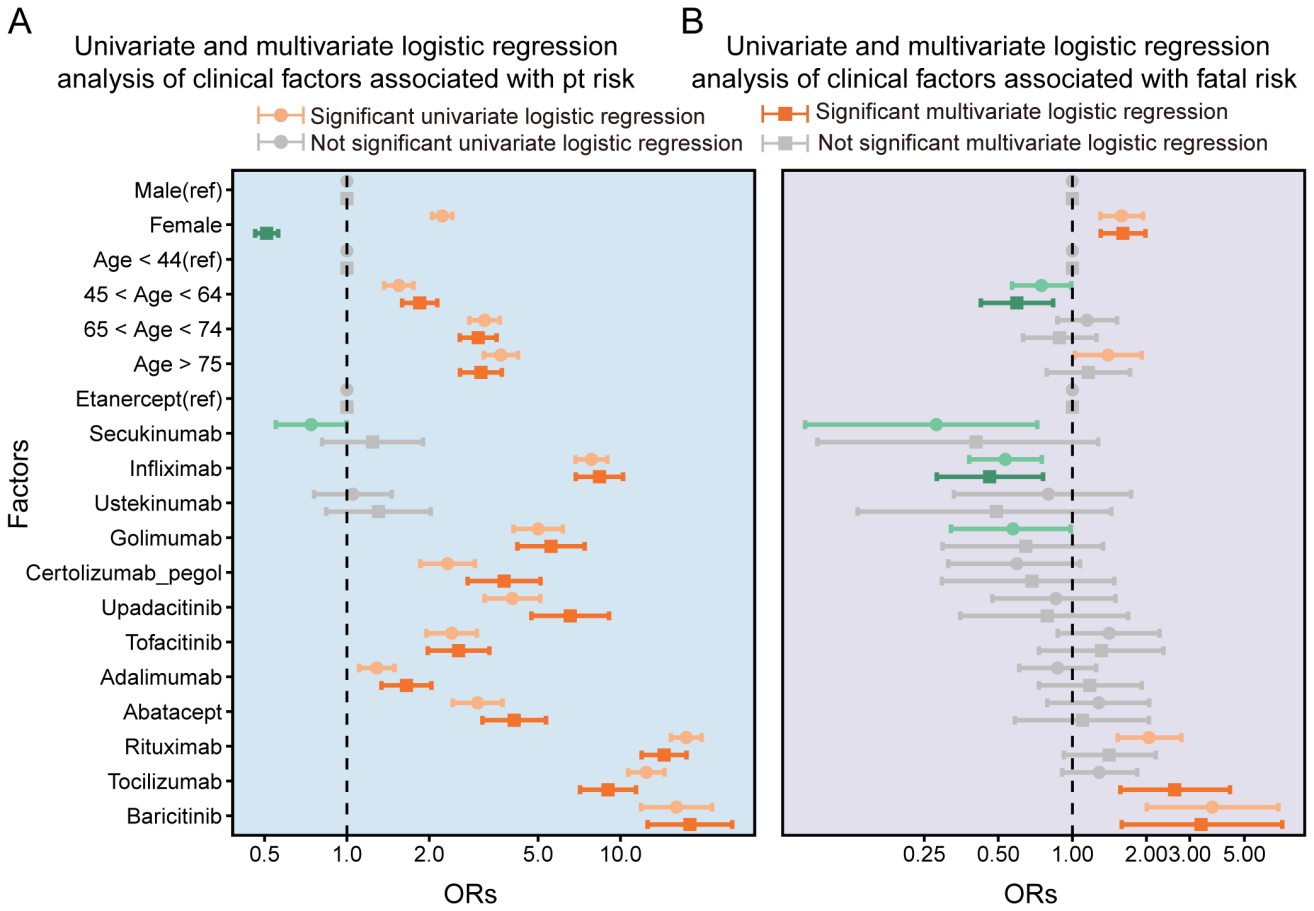
Univariate and multivariate logistic regression analysis of clinical factors associated with pt risk(A) and Univariate and multivariate logistic regression analysis of clinical factors associated with fatal risk.

Univariate logistic regression analysis indicated a significantly higher risk of PFI in females (OR = 2.23, 95% CI: 2.06–2.41, *p* < 0.001), but this association was not significant in the multivariate analysis. Both univariate and multivariate logistic regression analyses showed higher risk of fatal outcomes among females (OR = 1.67, 95% CI: 1.33–2.10, *p* < 0.001). Additionally, age emerged as a critical determinant of PFI risk. Patients aged ≥75 years exhibited a substantially elevated risk of PFI in both analyses: univariate OR = 1.40 (95% CI: 1.02–1.91, *p* < 0.05) and multivariate OR = 3.65 (95% CI: 3.16–4.22, *p* < 0.001). Among the drug types analyzed, nine biologics showed higher disproportionality in PFI reporting. Baricitinib demonstrated the highest risk: univariate OR = 3.71 (95% CI: 2.01–6.89, *p* < 0.001); multivariate OR = 17.95 (95% CI: 12.57–25.63, *p* < 0.001). It was also associated with the highest risk for fatal outcomes, and this association was statistically significant (univariate OR = 3.34; 95% CI: 1.59–7.13, *p* < 0.01). Rituximab was associated with a significantly higher risk: multivariate OR = 2.05 (95% CI: 1.53–2.77, *p* < 0.001) for PFI and multivariate OR = 14.43 (95% CI: 11.95–17.44, p < 0.001). Infliximab showed a significantly higher risk for PFI in multivariate logistic regression analysis (OR = 8.38, 95% CI: 6.87–10.22, *p* < 0.001). In multivariate analysis, tocilizumab was associated with a significantly higher risk of fatal outcomes (OR = 2.60, 95% CI: 1.57–4.37, *p* < 0.001). These results are presented in **Figure 5**.

### 3.5 Time-to-onset analysis

**Figure 6:**
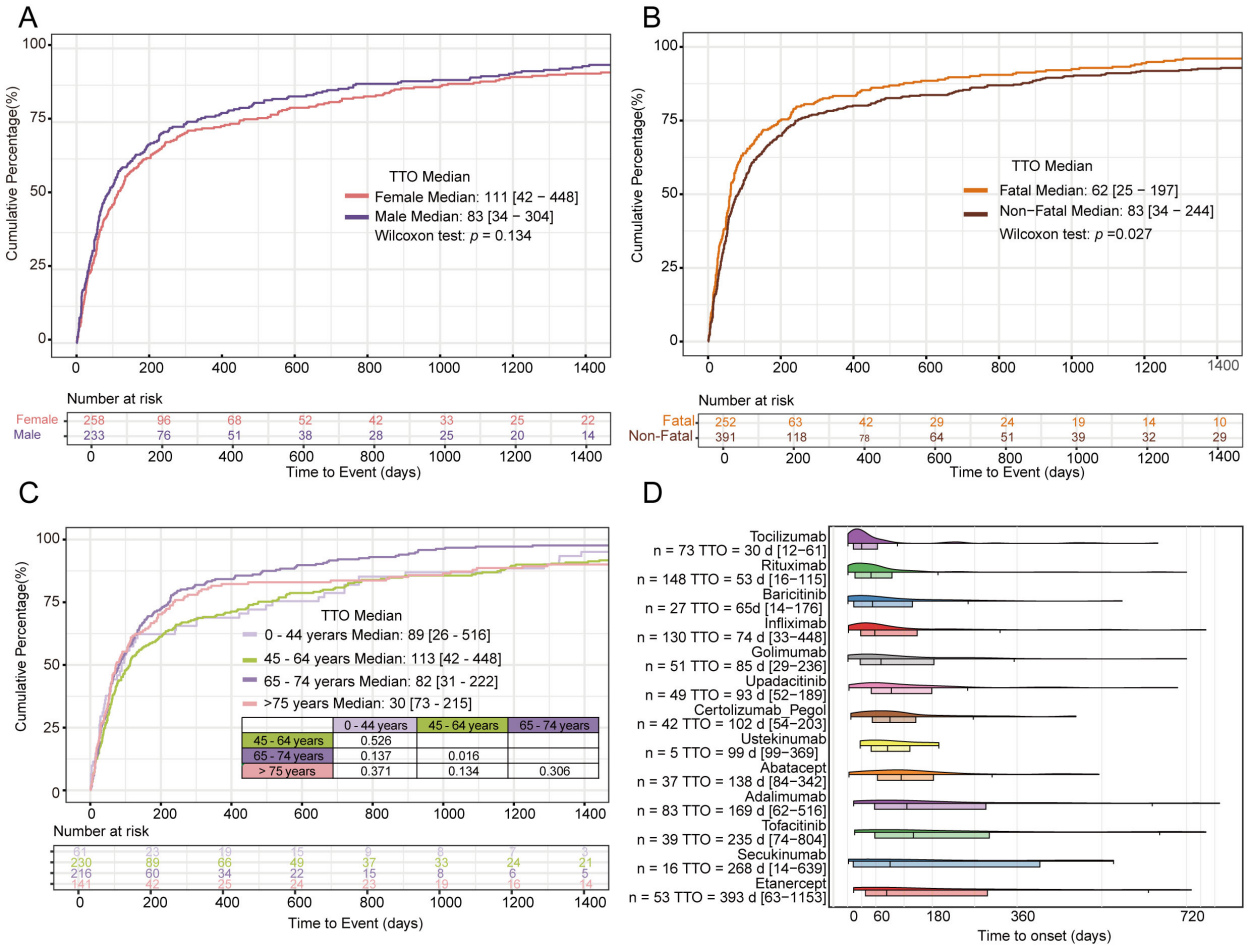
TTO analysis of gender **(A)** TTO analysis of fatal vs non-fatal **(B)** TTO analysis of age **(C)** TTO of different biological agents **(D)**.

The median TTO was 83 days (IQR 34–304) for males and 111 days (IQR 42–448) for females, with no statistically significant difference between genders (*p* = 0.134). This finding is presented in **Figure 6A**. The median TTO in the non-fatal group was 83 days (IQR 34–244), significantly longer than in the fatal group (62 days, IQR 25–197; *p* = 0.027). This comparison is shown in **Figure 6B**. The median TTO across age groups was: 0–44 years: 89 days (IQR 26–516); 45–64 years: 113 days (IQR 42–476); 65–74 years: 82 days (IQR 31–222). A significant difference in TTO was observed between the 45–64 years and 65–74 years groups (*p* = 0.016). For patients aged ≥75 years, the median TTO was 73 days (IQR 30–235), showing no statistically significant difference compared to the 45–64 years group. These results are displayed in **Figure 6C**.

Analysis of TTO across different biologics revealed a time-dependent gradient. The median TTO varied substantially: tocilizumab (30 days; IQR 12-61), rituximab (53 days; IQR 16-116), secukinumab (268 days; IQR 14-639), and etanercept (393 days; IQR 63-1153). Pairwise comparisons of TTO between biologics are provided in **Supplementary Table S3**. These findings are presented in **Figure 6D**.

## 4 Discussion

This large real-world study found that biologics were associated with the greatest disproportionality in reports of PFI-related AEs. Based on FAERS database reports from 2004 to 2024, approximately 0.19% of patients treated with biologics experienced PFI events.

Infliximab and rituximab, in particular, showed the strongest signals based on their elevated ROR. Conversely, our analysis identified a signal of an increased risk of fatal outcome for tocilizumab and baricitinib. As these four biologics represent distinct classes of immunomodulatory drugs, we focused our comparative safety analysis on them. The most prominent signal was observed for infliximab in association with pulmonary histoplasmosis. Notably, elevated risks were also identified for PJP, aspergillosis, and cryptococcal pneumonia with it. t. Histoplasmosis emerged as the most prevalent invasive fungal infection, with over 50% of cases linked to infliximab use (21). TNFi compromises host defences against Aspergillus fumigatus, thereby increasing aspergillosis risk (22). Among the biologics analysed, rituximab demonstrated the highest disproportionality in reports of PJP and aspergillosis. As a chimeric anti-CD20 monoclonal antibody, rituximab exerts its therapeutic effect through complement-dependent cytotoxicity and antibody-dependent cell-mediated phagocytosis of B cells (23, 24). However, this B-cell depletion concomitantly increases susceptibility to opportunistic infections. Emerging evidence indicates that B cells play crucial roles in maintaining T-cell-mediated immunity, and their depletion may consequently compromise host defences against pathogens, including Pneumocystis jirovecii (25, 26). Clinical studies report a 2.96% incidence of PJP among rheumatic patients receiving rituximab therapy (27). In our analysis, a strong disproportionality signal was observed for rituximab, which constituted 31.5% (n=1164) of all reports. PJP manifests with particularly severe clinical presentations in rheumatic disease patients, with reported mortality rates exceeding 40% (28).

Tocilizumab, an interleukin-6 (IL-6) receptor antagonist, demonstrated the strongest association with fungal pneumonia, specifically COVID-19-associated pulmonary aspergillosis (CAPA) and cryptococcosis (29, 30). Key risk factors for invasive pulmonary aspergillosis (IPA) include advanced age, chronic pulmonary disease, neutropenia, hematological malignancy; receipt of an allogeneic stem-cell transplant or solid organ transplant; use of immunosuppressants (e.g., calcineurin inhibitors, TNFi), ICU admission, mechanical ventilation (MV), and treatment with antibacterial agents, corticosteroids, or IL-6 receptor antagonists (31–33). The most prominent signals for cryptococcal pneumonia and aspergillosis were observed with baricitinib (a JAKi) among the biologics studied. Its mechanism of action involves modulating T cells, natural killer (NK) cells, and dendritic cells. Fungal infection patterns correlate with specific immune deficits: neutrophil dysfunction predisposes to mold infections (e.g., aspergillosis), while T-cell impairment increases susceptibility to infections caused by cryptococcus and pneumocystis jirovecii (34).

Our analysis observed a trend toward increased PFI risk in female patients, which may be associated with a higher likelihood of fatal outcomes. This sex-specific risk profile may be mediated by age-related declines in estrogen levels and the higher prevalence of vitamin D deficiency among female patients. Estrogens enhance humoral immunity by stimulating B-cell activity, promoting Th2 polarization, and prolonging the survival of autoreactive B and T lymphocytes (35). Estrogen levels decline progressively with advancing age. Compelling evidence demonstrates that vitamin D deficiency elevates susceptibility to respiratory infections. Specifically, vitamin D deficiency impairs host defenses against *Aspergillus fumigatus* by exacerbating and prolonging inflammatory responses (36, 37). The observed disproportionality signal with advancing age is consistent with the established understanding of age as a risk factor. This age-associated susceptibility is exacerbated by immunosenescence and progressive physiological deterioration (38). Immunosenescence, a complex immunological remodeling process associated with aging, correlates with elevated infection risk and increased all-cause mortality (39). Furthermore, multiple comorbidities in elderly patients increase PFI susceptibility, including COPD, advanced lung cancer (especially chemotherapy-treated cases), diabetes mellitus (DM), solid organ malignancies, and chronic kidney disease (CKD) (40).

Our analysis identified a signal of an elevated risk for PFIs with these nine biologics. Our findings suggested an association between tocilizumab and baricitinib and an increased risk of fatal outcome. It is plausible that the observed increase in mortality might be, in part, attributable to the known effects of tocilizumab and baricitinib on neutrophil depletion and T-cell impairment—both established risk factors for PFIs. In our research, tocilizumab and baricitinib were associated with signals of disproportionate reporting for cryptococcal pneumonia, bronchopulmonary aspergillosis, and mucormycosis. PJP, pulmonary aspergillosis, histoplasmosis, coccidioidomycosis, and mucormycosis are life-threatening opportunistic infections in immunocompromised individuals and remain a significant global health concern. Despite effective chemoprophylaxis, current estimates indicate up to 500,000 annual cases globally, with mortality rates ranging from 10% to 50% (41–43). The B-cell-depleting effect of rituximab persists for 6 to 12 months. A cohort study found that approximately 80% of PJP cases in rituximab-treated patients occurred within six months of initiating or intensifying immunosuppressive therapy (44). A previous study demonstrated an association between tocilizumab use and *Aspergillus* coinfection with increased mortality risk (37). A multicenter study revealed that patients with cryptococcosis were nearly 19 times more likely to have received tocilizumab compared to those without the infection (30). Additionally, higher rates of cryptococcosis were observed in COVID-19 patients with comorbidities, including heart failure, type 2 diabetes mellitus, and CKD. Notably, CD4+ T-cell counts were significantly lower in COVID-19 patients who developed cryptococcosis, suggesting that impaired cell-mediated immunity may contribute to the pathogenesis of this opportunistic invasive fungal infection (IFI) (30). Invasive mucormycosis is a life-threatening fungal infection that predominantly affects immunocompromised patients with underlying comorbidities (32). A previous study reported that mortality rates for pulmonary mucormycosis range from 48% to 87% (45).

Our analysis revealed no statistically significant difference in median TTO between male and female patients. The median TTO was significantly shorter in the fatal group (62 days; IQR 25-197) compared to the non-fatal group (83 days; IQR 34-244; p=0.027). These findings underscore the need for clinical vigilance regarding PFI onset timing. The median TTO varied significantly across age groups, with the shortest duration observed in patients aged ≥75 years (73 days; IQR 30-235). A pronounced temporal gradient in median TTO was observed among biologics, ranging from 30 days (tocilizumab) to 393 days (etanercept). The median TTO for infliximab in our study was 74 days (IQR33-448), differing significantly from the 55 days reported previously (46). Rituximab exhibited a median TTO of 53 days (IQR 16-116), significantly shorter than the 84 days reported in prior studies (47). In our analysis, tocilizumab was observed to have the shortest median TTO (30 days; IQR 12-61), with clinical manifestations frequently emerging shortly after treatment initiation.

Among the top 30 AEs associated with biologics, infectious pneumonia and bacterial pneumonia were the most frequently reported. Other commonly reported infections included cytomegalovirus infection and herpes zoster, consistent with previous research findings. Respiratory-related adverse events—including respiratory failure, dyspnea, interstitial lung disease (ILD), and acute respiratory distress syndrome (ARDS)—frequently required MV. Additionally, neutropenia was a notable adverse event. Both mechanical ventilation and neutropenia are established risk factors for pulmonary aspergillosis. Sepsis and aspergillosis were also common concurrent adverse events.

The advent of next-generation sequencing (NGS) and other advanced diagnostic modalities has heightened clinical awareness of PFIs associated with biologics. Our disproportionality analysis suggested potential associations between PFIs and several biological agent classes: TNFi, IL-6i, IL-17i, IL-12/23i, IL-23i, JAKi, CTLA4-Ig, and B-cell depleting agents, using other drugs in the FAERS database as comparators. These findings provide important insights into the associations between biologics and the risks of various PFIs.

According to the Infectious Diseases Society of America (IDSA) guidelines for immunocompromised hosts, management of patients developing endemic fungal infections during TNFi therapy should include: (1) discontinuation of the TNFi; and (2) prompt initiation of antifungal therapy with polyenes (e.g., amphotericin B) or azoles (e.g., itraconazole, fluconazole) (48). Substantial evidence indicates that trimethoprim-sulfamethoxazole (TMP-SMX) prophylaxis significantly reduces mortality in this vulnerable population (49, 50). Reflecting this evidence, the current American College of Rheumatology/Vasculitis Foundation guideline for managing antineutrophil cytoplasmic antibody (ANCA)-associated vasculitis (AAV) provides a conditional recommendation for PJP prophylaxis during rituximab therapy (51). In 2021, the European Crohn’s and Colitis Organisation (ECCO) strongly recommended primary PJP prophylaxis with TMP-SMX for inflammatory bowel disease (IBD) patients receiving triple immunosuppressive therapy (e.g., corticosteroids, methotrexate, thiopurines, or biologics). Prophylaxis may also be considered for patients on dual immunosuppression, particularly with calcineurin inhibitors or additional risk factors (e.g., high-dose corticosteroids, lymphopenia, or JAKi use) (52). Baricitinib, golimumab, infliximab, tocilizumab, abatacept, certolizumab pegol, upadacitinib, and tofacitinib are associated with an elevated risk of PJP. Prophylactic treatment with TMP-SMX should be considered when using these biologics to mitigate infection risk. Female patients, elderly individuals, and those with chronic conditions (e.g., CKD, heart failure, DM) require close monitoring for PFI risk. Thirteen biologics showed the highest disproportionality in reporting of PFIs. The FDA has issued black box warnings for infliximab, certolizumab pegol, golimumab, adalimumab, etanercept, tofacitinib, upadacitinib, tocilizumab, and baricitinib, indicating an elevated risk of severe infections potentially leading to hospitalization or death. The biological agent should be discontinued if a patient develops severe infection or sepsis. Empirical antifungal therapy should be considered for patients with severe systemic diseases at high risk of IFIs. Patients receiving these agents require close clinical monitoring for signs and symptoms of infection during and after treatment (53). Due to the elevated risk, close monitoring for fatal fungal infections is essential during tocilizumab or baricitinib treatment.

Our clinical recommendations derive from disproportionality analyses using ROR to evaluate associations between various biologics and PFIs in real-world adverse event reports. These findings may inform clinical decision-making regarding biological agent use and facilitate anticipation of potential adverse drug reactions (ADRs). Despite their substantial therapeutic benefits, the potential for severe PFIs needs to be considered with biologic therapies. Therefore, their clinical application requires rigorous risk-benefit evaluation prior to initiation.

Our study observed associations between biologics and PFIs, suggesting a potential link. However, several limitations of our study should be acknowledged. First, the FAERS database, as a passive surveillance system, is inherently subject to reporting biases, including substantial under-reporting and selective reporting. Since reporting is voluntary, the volume of captured reports does not represent the true incidence of adverse events and is influenced by extraneous factors such as a drug’s market longevity, media exposure, and the perceived severity of the event. This framework creates a potential for differential reporting bias among different biologics. Newer agents or those with “black box” warnings, for example, are often subject to heightened scrutiny and increased reporting (the Weber effect), in contrast to older, more established therapies. A drug’s media attention, approved indications, and specific patient population can further distort reporting rates. Therefore, while significant, the disproportionality signals we identified may be confounded by these reporting artifacts and do not solely represent genuine differences in safety profiles. Second, incomplete patient information (e.g., comorbidities and concomitant medications) prevents definitive assessment of their potential influence on biological agent-associated PFI. Third, the critical limitation of the FAERS database is the lack of case-by-case clinical verification. This absence means that reports often contain incomplete or inaccurate data, and more importantly, it precludes any ability to establish causality, as ROR quantify association rather than risk or causation. A statistical signal from FAERS cannot determine if the drug actually caused the event or if it was due to the patient’s underlying disease or other treatments. Consequently, biases in unpredictable directions may be present. Therefore, these signals remain unvalidated hypotheses requiring confirmation through studies capable of individual patient assessment.

## 5 Conclusion

Our study was limited by inherent reporting bias in the FAERS database, and causality between biological agent use and PFIs could not be established. However, a significant association was observed between biologics and PFI based on disproportionality analysis. Infliximab, rituximab, tocilizumab, and baricitinib exhibited higher disproportionality reporting signals for PFIs compared to other biologics. A trend toward a lower risk of PFIs was observed with etanercept compared to other biologics in the study. We advise that it is essential to weigh the benefits and risks comprehensively before starting biological therapy. Initiating antifungal prophylaxis may be warranted for patients on high-risk biologics, especially when other risk factors are present.

## Data Availability

The original contributions presented in the study are included in the article/**Supplementary Material**. Further inquiries can be directed to the corresponding author.
