# Supplementary material for: Risk of pulmonary fungal infections associated with biologics: a FAERS database disproportionality analysis
Source: Front Immunol. 2025 Oct 20;16:1672343. doi: 10.3389/fimmu.2025.1672343 (PMC12580261; doi:10.3389/fimmu.2025.1672343)
Supplement: Supplementary file 1 [file Table1.docx]

Supplementary Material

## Table 1. Search terms for biologics treating rheumatoid or psoriasis approved by FDA

| **Class** | **Generic name** | **Approved date** |
| --- | --- | --- |
| TNF-α inhibitors | Adalimumab | 12/31/2002 |
|  | Certolizumab_pegol | 4/22/2008 |
|  | Etanercept | 11/2/1998 |
|  | Golimumab | 4/24/2009 |
|  | Infliximab | 8/24/1998 |
| IL-6 inhibitors | Tocilizumab | 1/8/2010 |
| IL-17 inhibitors | Secukinumab | 1/21/2015 |
| IL-12/23 inhibitors | Ustekinumab | 9/25/2009 |
| JAK inhibitors | Baricitinib | 3/31/2018 |
|  | Upadacitinib | 8/16/2019 |
|  | Tofacitinib | 11/6/2012 |
| T-cell co-stimulation inhibitors | Abatacept | 12/23/2025 |
| B-cell depleting agents | rituximab | 11/26/1997 |

## Table 2. pulmonary fungal infection categorization according to Medical Dictionary for Regulatory Activities (MedDRA) Classification Version 25.1

| **Group** | **MedDRA terms included** |
| --- | --- |
| *Histoplasma* | Acute pulmonary histoplasmosis(PT 25.1) |
|  | Pulmonary histoplasmosis |
| *Aspergillosi* | Bronchopulmonary aspergillosis(PT 25.1) |
|  | Pneumonia fungal(PT 25.1) |
| *Cryptococcus* | Coccidioidomycosis(PT 25.1) |
| *mucormycosis* | Disseminated mucormycosis(PT 25.1) |
|  | Mucormycosis(PT 25.1) |
|  | Pulmonary mucormycosis |
| *Candida* | Candida pneumonia(PT 25.1) |
|  | Respiratory moniliasis(PT 25.1) |
| *Pneumocystis jirovecii* | Pneumocystis jirovecii pneumonia |
|  | Pneumocystis jirovecii infection |
| *cryptococcal* | Pneumonia cryptococcal |

**Table 3. comparisons of TTO between biologics**

|  | Rituximab | Adalimumab | Etanercept | Abatacept | Certolizumab_pegol | Golimumab | Tofacitinib | Tocilizumab | Secukinumab | Ustekinumab | Baricitinib | Upadacitinib |
| --- | --- | --- | --- | --- | --- | --- | --- | --- | --- | --- | --- | --- |
| Infliximab | 0.014 | 0.007 | 0.004 | 0.006 | 0.202 | 0.528 | 0.033 | <0.001 | 0.287 | 0.405 | 0.125 | 0.183 |
| Rituximab |  | <0.001 | <0.001 | <0.001 | <0.001 | 0.055 | <0.001 | 0.0002 | 0.019 | 0.123 | 0.566 | 0.012 |
| Adalimumab |  |  | 0.07 | 0.859 | 0.047 | 0.047 | 0.482 | <0.001 | 0.144 | 0.984 | 0.024 | 0.062 |
| Etanercept |  |  |  |  | 0.001 | 0.001 | 0.488 | <0.001 | 0.247 | 0.564 | 0.002 | <0.001 |
| Abatacep |  |  |  |  | 0.198 | 0.04 | 0.133 | <0.001 | 0.275 | 0.997 | 0.038 | 0.07 |
| Certolizumab_pegol |  |  |  |  |  | 0.442 | 0.013 | <0.001 | 0.05 | 0.704 | 0.148 | 0.761 |
| Golimumab |  |  |  |  |  |  | 0.037 | <0.001 | 0.073 | 0.389 | 0.517 | 0.687 |
| Tofacitinib |  |  |  |  |  |  |  | <0.001 | 0.166 | 0.768 | 0.029 | 0.01 |
| Tocilizumab |  |  |  |  |  |  |  |  | 0.004 | 0.013 | 0.021 | <0.001 |
| Tocilizumab |  |  |  |  |  |  |  |  |  | 0.871 | 0.055 | 0.07 |
| Secukinumab |  |  |  |  |  |  |  |  |  |  | 0.399 | 0.781 |
| Ustekinumab |  |  |  |  |  |  |  |  |  |  |  | 0.146 |
| Baricitinib |  |  |  |  |  |  |  |  |  |  |  |  |
